# Sentinel Lymph Node Biopsy in Nonmelanoma Skin Cancer Patients

**DOI:** 10.1155/2013/267474

**Published:** 2013-02-14

**Authors:** Marie-Laure Matthey-Giè, Ariane Boubaker, Igor Letovanec, Nicolas Demartines, Maurice Matter

**Affiliations:** ^1^Department of Visceral Surgery, University Hospital CHUV, Lausanne, Switzerland; ^2^Department of Nuclear Medicine, University Hospital CHUV, Lausanne, Switzerland; ^3^Department of Pathology, University Hospital CHUV, Lausanne, Switzerland

## Abstract

The management of lymph nodes in nonmelanoma skin cancer patients is currently still debated. Merkel cell carcinoma (MCC), squamous cell carcinoma (SCC), pigmented epithelioid melanocytoma (PEM), and other rare skin neoplasms have a well-known risk to spread to regional lymph nodes. The use of sentinel lymph node biopsy (SLNB) could be a promising procedure to assess this risk in clinically N0 patients. Metastatic SNs have been observed in 4.5–28% SCC (according to risk factors), in 9–42% MCC, and in 14–57% PEM. We observed overall 30.8% positive SNs in 13 consecutive patients operated for high-risk nonmelanoma skin cancer between 2002 and 2011 in our institution. These high rates support recommendation to implement SLNB for nonmelanoma skin cancer especially for SCC patients. Completion lymph node dissection following positive SNs is also a matter of discussion especially in PEM. It must be remembered that a definitive survival benefit of SLNB in melanoma patients has not been proven yet. However, because of its low morbidity when compared to empiric elective lymph node dissection or radiation therapy of lymphatic basins, SLNB has allowed sparing a lot of morbidity and could therefore be used in nonmelanoma skin cancer patients, even though a significant impact on survival has not been demonstrated.

## 1. Introduction

20 years ago [1], sentinel lymph node biopsy (SLNB) was introduced for melanoma patients and later for numerous other tumors with lymphatic metastatic propensity. Even though surgical oncology community is divided in believers and nonbelievers regarding its application, data show that SLNB has already changed the treatments modalities in melanoma and breast cancer patients, at least with respect to TNM classification. It has allowed a better understanding of disease progression and response to treatment in patients with comparable staging groups. 

Nonmelanoma skin cancer with potential metastatic spreading to regional lymph nodes regroups skin lesions like high-risk squamous cell carcinoma (SCC), Merkel cell carcinoma (MCC), and pigmented epithelioid melanocytoma (PEM). Because of the low incidence of nonmelanoma skin cancer with potential metastatic spread and the lack of large clinical trials, the use of SLNB in these cases is not well established, and no guidelines are currently available. Previous studies conducted about this subject reported a high rate of positive sentinel nodes (SNs) in nonmelanoma skin cancer: 4.5–28% for SCC [2–4], 16–42% for MCC [5–7], and 14–46% for PEM [8, 9]. In this context, the role of SLNB in nonmelanoma skin cancer should be accepted as a standard staging procedure assuming that N status is a strong predictive factor for survival.

## 2. Material and Methods

Over a 10-year period from January 2002 to December 2011, a total number of thirteen patients underwent a SLNB for nonmelanoma skin cancer at the University Hospital of Lausanne, Switzerland. The patients were identified and registered in parallel of a melanoma patients' registry (550 SLNB during the same time), and a retrospective analysis was performed. Data were retrieved form patient's files and imaging database. The study protocol and data collection were approved by our audit department. 

Based on established protocol published earlier [10], a triple technique was used to identify SNs. Briefly, a 99^m^ Tc nanocoll lymphoscintigraphy was performed preoperatively (day before or same day), followed by intraoperative injection of 2 mL. patent blue V intradermally around the primary tumor or in the scar. SN was localized using hand-held gamma-probe guidance. Histopathology followed standardized analysis for melanoma patients: serial sectioning, H.-E, and corresponding immunohistochemistry (S100 protein and Melan-A or cytokeratins)

Data retrieved included demographics, type of primary tumor, number of SN removed, number of positive SNs, completion lymph node dissection (CLND), nonsentinel lymph nodes (NSNs), and oncological followup including local recurrence, lymphatic extension, metastasis, disease-free, and overall survival.

The aim of our present study was to analyze the rate of positive SNs and reliability regarding false negative rate during followup in nonmelanoma skin cancer patients in our series and compare our data with a review of the current literature.

## 3. Results

### 3.1. Patient's Demographics

Thirteen patients with nonmelanoma skin cancer underwent SLNB. Of these, eight presented a squamous cell carcinoma (SCC), three a Merkel cell carcinoma (MCC), and two a pigmented epithelioid melanocytoma (PEM). The median age was 68 years (range 25–92 years; mean ± SD: 61.5 ± 21.2). There were 8 males and 5 females. Clinical details are summarized in Table 1. 

### 3.2. Biopsy Results, Outcomes of Surgery, and Followup

The rate of positive SN for the 13 patients was 31% (4/13). The median followup was 23 months (range 2–76 months). Fifty SNs were removed, and 7 were positive in 4 patients. 

Patient 1 with MCC of right buttock had one micrometastatic (<2 mm deposit) in 4 examined SNs. Radiation therapy was given to the buttock and inguinal areas. Patient 4 with MCC of the left leg had one metastatic SN (6 mm) out of 2. She refused any further adjuvant therapy. Both MCC N+ patients are free of recurrent disease with a follow-up period of 20 and 54 months, respectively.

Patient 7 with a high-risk SCC of left thumb had 2 metastatic SNs (1.5 cm and 1 cm) out of 3. Two deep suspicious nonsentinel nodes (NSNs) were also removed during the same intervention, and one showed a 3 cm metastasis. The patient was preoperatively investigated only by MRI of the arm, and clinically no axillary node was palpable. ELND was performed, and 2 NSNs out of 10 were positive. He had adjuvant radiation therapy (50 Gy) and chemotherapy (carboplatin) following axilla recurrence. Unfortunately the patient ultimately needed an amputation with disarticulation 15 months after the diagnosis because of further progression of the disease in the axilla. He is still alive 23 months after the diagnosis.

Patient 11 with a deep (11 mm) PEM of the middle of the back had SLNB in both axillas. On the right side, 1 SN showed only one capsular focus of metastatic cells and following a second opinion of an international expertise center we decided not to proceed with ELND. One NSN was negative. On the left side, one SN had a parenchymal focus of PEM and was considered positive. Three other NSNs were negative and ELND showed 12 other negative NSNs. She had no evidence of disease within a followup of 5 years.

Nine patients were found to be SN negative. Seven patients had SCC: patients 6 and 13 were lost during the followup, after 2 and 6 months, respectively. Patient 2 with epidermolysis bullosa developed other SCC lesions on the upper and lower limbs 9 months, respectively 4 years after the excision of the primary tumor of the lower limb. Patient 5 with an initial SCC of the right vulva underwent an excision of the contralateral vulva for a VIN 3 tumor 3 years later. Patient 8 died from an aggressive locoregional progression of the disease six months after the diagnosis. Patients 9 and 10 showed no recurrence after a followup of 9 and 15 months. Patient 12 with PEM was lost during followup after 11 months, and patient 3 with MCC did not recur after a followup of 6 years.

**Figure 1:**
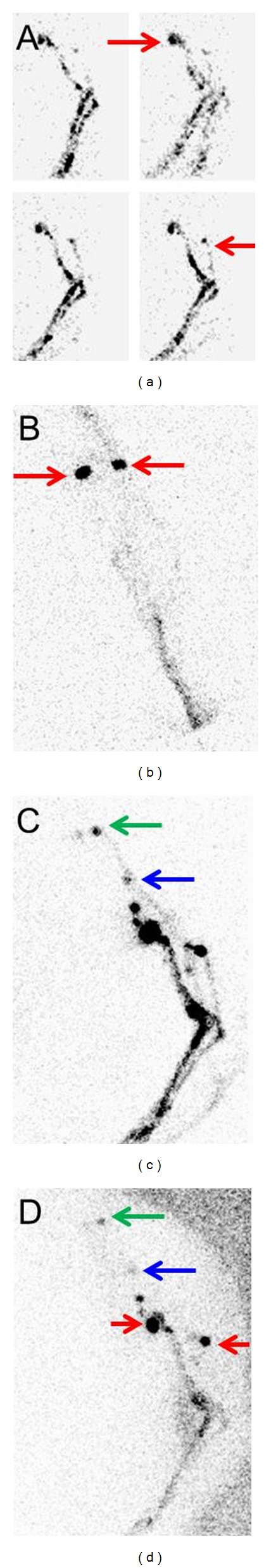
Patient 13 had a 3.5 cm large poorly differentiated SCC on the dorsal side of left hand that was reaching subcutaneous level with perineural invasion but with no lymphovascular invasion. Dynamic lymphoscintigraphy of the upper left limb demonstrated multiple drainage pathways on the dynamic views (a), and accessories lymph nodes were immediately visualized in the humeral lateral and medial regions (red arrows). These were confirmed not to be only ectatic lymphatic vessels but 2 different sentinel nodes corresponding to 2 basins (b). Delayed views of the arm and shoulder (c, d) showed 2 more SNs in 2 basins: in the axilla (blue arrow) and basicervical (green arrow). All 7 SNs in 4 basins were negative.

Patient 13 presented a 3.5 cm large poorly differentiated SCC, and lymphoscintigraphy identified four different SN basins (3 interval nodes): humeral lateral, and medial, axilla and cervical (Figure 1). All 7 SNs were negative.

Overall no postoperative complication at the SLNB site was registered. No patient with negative SN had a nodal recurrence.

## 4. Discussion

Our experience confirms results of other series regarding feasibility and reliability (false negative rate) of SLNB. This cohort of patient with heterogeneous group of rare primary skin carcinomas reflects the experience in the literature. The rate of 30.8% positive SNs observed in the current study is comparable to those of similar studies published on non-melanoma skin cancer patients. For instance, Cecchi et al. [18] and Wagner et al. [19] reported a rate of positive SNB of 20% (2/10) and 31.8% (7/22), respectively. Of note, majority of series describe a pretty limited number of patients. In our own study, patients who were SN positive or negative had no nodal recurrence and disease recurred or progressed regionally independently of SN status (one patient in each group of SN positive or SN negative). 

Following potential advantages of SNB must be underlined.

1. Detection of regional lymph node basins at risk for N+ status: lymphatic mapping using lymphoscintigraphy is useful in defining lymphatic basins at risk, which is very important in complex lymphatic network in head and neck surgery for example [20].
2. Staging of real N0 patients, in whom unnecessary CLND or radiation therapy and their significant morbidity can be spared, meaning that false negative assessment (CLND or node recurrence during follow-up) must be as low as possible. It should be assumed that removing early metastatic node improves significantly the prognosis compared with removing advanced nodal disease.
3. Detection of metastatic and micrometastatic diseases in clinically and radiologically N0 patients. Overstaging is possible (uncertain meaning of isolated tumor cells), but false negative N0 patients (clinically negative and H-E negative) can be detected and their staging is correctly assessed for directing appropriate treatment.
4. Detection of interval SNs (lymph nodes outside usual basins) that are at the same metastatic risk as other SNs and that can be otherwise misinterpreted as in-transit metastases [10, 17, 21–23] (Figure 1).

On the other hand SNLB has a price: a hospital stay and surgery and its own morbidity. Adverse effects of SLNB were observed in 25% compared with 70% with SLNB and CLND in the Z0011 breast cancer trial (6% lymphedema versus 11% at one year) [24]. In the Sunbelt Melanoma Trial overall morbidity was 4.6% for SLNB alone compared with 23.2% for SLNB and CLND. Lymphedema following SLNB was 0.3% in the axilla and 1.5% in the groin [25]. Incidence of adverse reactions to different blue dyes used for SLNB is 1–3% [26].

## 5. Squamous Cell Carcinoma (SCC)

SCC represents the second most frequent skin cancer after melanomas [3, 4] and its incidence in the population reaches approximately 1% [4]. Incidence of SCC varies widely according to patients' risk like sun exposure or immunosuppression. Most of these patients will not develop nodal disease, but in some patients it represents the first metastatic step. The reported metastatic rate of high-risk SCC reaches 11–47.3% [27], and the regional lymph nodes are the first involved. Patient with clinical detectable nodal metastasis has a poor prognosis with a reported 5-year survival rates of 26% [27].

Risk factors for metastasis or local recurrence of SCC have been described in the literature and are summarized in Table 2 [19, 28–31]. They should be used to select patients eligible for SLNB. Despite the absence of controlled studies, guidelines about the staging for high-risk SCC in immunosuppressed patients or patients planned for a transplantation recommend to perform a SLNB [32]. 

Recently the French Dermatology Recommendations Association (aRED) suggested a prognostic classification including 2 groups defined as low-versus-significant metastatic risk [33]. Unlike previously published guidelines reviewed by Veness [29] that proposed no recommendation for the management of lymph nodes in high-risk patients, aRED stated that SLNB may be envisaged for clinical trials and evaluation studies. Their proposal was ultrasound surveillance and no routine ELND or radiation therapy [33].

The low rate of false negative SLNB reported in the literature [2, 34] is an essential quality marker for SLNB efficacy. False negative rate seems to depend on SCC location: all sites 15.4%, head and neck 0% and truncal/extremity 22.2% [35]. In the absence of consensus in high-risk SCC patients, 46% of surgeons proposed SLNB in Jambsaria-Pahlanjani's survey [36]. 

They are only a few studies published about high-risk patients presenting SCC arising from a burn scar or a chronic ulcer (Marjolin's ulcer) [22, 37] (patient 8), locally recurrent SCC [38], and patients with recessive-type epidermolysis bullosa (patient 2) [39]. 

## 6. Merkel Cell Carcinoma (MCC)

MCCs are rare and aggressive neuroendocrine tumors arising from cutaneous Merkel cells. Their incidence seems to be rising; they affect more elderly and immunosuppressed people with a correlation to sun exposure. They tend to spread locally before developing distant metastasis, and at time of diagnosis up to 68% of patients already present lymph node involvement [40]. The presence of clinically palpable nodes and visible lymphadenopathy on CT scan is an indicator of poor survival rate [13] so that early detection of lymph node involvement is the most important prognostic factor. 

MCC is known to be radiosensitive, but the systematic use of radiotherapy to the primary tumour and/or the lymph node basin is still debated. Eich et al. [41] have reported a significant higher disease-free survival rate and Mojica et al. have reported [42] a higher overall survival rate after adjuvant radiotherapy. However, Allen et al. could not demonstrate that an adjuvant radiotherapy was necessary if the primary tumor and the lymph node basin were surgically controlled (ELND, SLNB, and CLND) [5]. Conversely in patient without nodal control (SLNB/ELNB) metastatic lymph node will appear in 45% of cases and radiotherapy is mandatory.

Already in 2002, Goessling et al. listed 49 patients with MCC and concluded that the SLNB could be a useful tool for their staging [43]. Since then, the use of SLNB for MCC has been the subject of several reviews which are summarized in Table 3 [5, 7, 11–17]. The cumulated rate of positive SNs was 31% (101/326). Only half of the SN positive patients underwent a CLND, and the rate of positive NSNs after CLND was 35% (19/54). It seems that despite the absence of guidelines, the number of patients undergoing SLNB followed by CLND is increasing. 

Criteria for a high risk of metastatic sentinel node in Merkel cell carcinoma are presented in Box 1. However, patients without these criteria still have a 23–36% risk for positive SN [41, 42, 49].

One of the largest monocentric study was presented by Fields et al. [16]. From 153 patients who underwent SLNB, 45 of them presented positive SN. CLND was consecutively performed in 21 patients, and 6 of them presented metastatic NSNs. During a median followup of 41 months 8/99 SN-negative patients developed nodal recurrence which corresponds to a false negative rate of 15%. The presence of lymphovascular invasion (LVI) was highly predictive for the disease-free and overall survival but not for the SN status. Interestingly 71% of the patients with positive SN and 92% of the patients with negative SN did not receive any adjuvant therapy. In this study, the author recommends to perform routinely SLNB even by patients who are clinically staged as N0. However, this staging procedure remains a subject of controversies in the recent published studies [15, 44, 45]. 

The use of immunohistochemistry (pancytokeratin and CK-20 antibodies) can significantly upstage false negative SNs [46, 47] and should be the role for SN examination. 

In summary up to 68% of MCC patients present nodal metastases at time of diagnosis. 20–30% of clinically N0 patients can be upstaged if a SLNB is performed. Nodal status is an important prognostic factor. The exact role and benefit of radiation therapy on lymphatic basins are not definitively assessed (clinically negative or after SLND, CLND, and ELND) [5, 13, 14, 42]. New attempts for improving standardized histopathology report [48] and treatment algorithm [49] would be helpful. 

MCC has a higher incidence in transplanted patients. These patients are younger and their 5-year overall survival of 46% [50] is slightly lower than the 54% observed in a large MCC data base regarding matched population [51].

## 7. Nonmelanoma Pigmented Tumors

Some patients with Spitz naevi may present with a difficult differential diagnosis for other melanocytic tumors including melanoma. A review of the literature about spitzoid tumors showed that 37.7% of patients presented metastatic SNs, and 14% of the patients with CLND had metastatic NSNs [52]. Metastatic propensity will define malignancy, but in SN negative patients only the followup can exclude it. These results were published by Magro et al. who reviewed their experience with SLNB in borderline melanocytic tumors (BMTs) [9].

Pigmented epitheloid melanocytoma, also called equine or animal-type melanoma, is a rare melanocytic tumor with frequent metastatic spreading to local lymph nodes and occurs mainly during childhood and in young adults [8].

However, Mandal et al. observed that patients with positive SN had an excellent outcome. They concluded that, while sparing risk for progressive bulky metastatic lymph node, SLNB would not change the prognosis in this low-grade melanocytic neoplasm. As no high-risk PEM has been identified, simple surveillance of the lymphatic basin with ultrasound seems to be a safe solution [53]. 

## 8. Other Rare Skin Neoplasms

SLNB has also been evaluated for cutaneous apocrine adenocarcinoma [54] and for aggressive digital papillary adenocarcinoma [55]. As both of them have a high propensity for lymphatic invasion, the systematic use of SLNB should be recommended. 

Because of their lymph node metastatic risk, some soft tissue sarcomas may also been concerned by SLNB. Lymph node dissection is recommended in clinically or radiologically N+ patients [56], but the role of SNB has not been clearly established yet. 

Lymph node metastatic rate for epithelioid sarcoma and angiosarcoma ranges from 17 to 80% and 11 to 40%, respectively. 

Regarding skin lymphomas a large recent series of patients with mycosis fungoides and Sezary syndrome showed that 91% of patients are clinically N0 [57]. As TNM plays a role in prognosis and treatment, SLNB could find its place for detecting patients with early stages (IA-IB) who adversely progress. In cutaneous T-cell lymphoma, SLNB can prove the primary cutaneous origin and avoid a systemic treatment [58].

## 9. Conclusion

Management of high-risk nonmelanoma skin tumor patients is still a matter of debate in the absence of randomized trials. Randomized studies are difficult to conduct because of the rarity of these tumors; the best option is therefore to pool these patients in multicentric cohort in order to support the guidelines defined by consensus. Definition of high-risk parameters and standardization of examination protocols could allow such studies of these rare but life-threatening malignancies. SLNB has its price and has its own morbidity; it represents, however, the best way for assessing N stage in clinically and radiologically negative patients. It must be remembered that a definitive survival benefit with SLNB in melanoma patients has not been proven (yet) and is currently evaluated by the MSLT 2 trial. However, because of its low morbidity compared with empiric ELND or radiation therapy on lymphatic basins, SLNB has already spared a lot of morbidity in many patients, before this survival advantage can also be demonstrated in nonmelanoma skin cancer patients. Thus, until better data demonstrate the opposite, SLNB should be recommended in nonmelanoma skin tumors.

## Figures and Tables

**Box 1:**
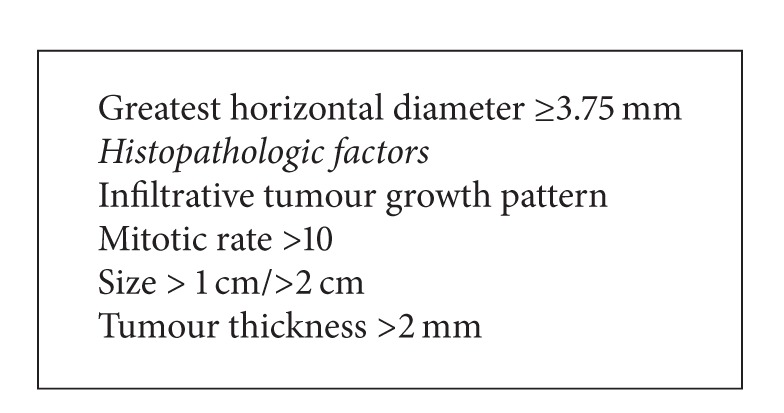
Criteria for a high-risk of metastatic sentinel node in Merkel cell carcinoma.

**Table 1:** Patient characteristics, sentinel lymph nodes results, and followup.

Patient	Age	Sex	Type	Primary site	SLN region	Risk factor	SLN total	SLN+	Clearance	Adjuvant therapy	Follow-up (months)	Recurrence
1	78	M	Merkel	Buttock	Inguinal	4 cm large	4	1	No	Yes	20	No
2	39	M	SCC	Leg	Inguinal	EBD	3	0	No	No	75	No
3	72	M	Merkel	Buttock	Inguinal	2.2 cm large	2	0	No	No	76	No
4	84	F	Merkel	Leg	Inguinal	LVI	4	1	No	No	54	No
5	33	M	SCC	Vulva	Inguinal Iliac	2 cm large 7 mm deep	3	0	No	No	52	No
6	92	F	SCC	Leg	Inguinal	5 cm large 7 mm deep	5	0	No	No	2	?
7	72	M	SCC	Thumb	Axillary	Recurrent, 3 cm large Bone infiltration	6	3	Yes	Yes	23	Yes
8	51	F	SCC	Thigh	Iliac	Chronic scar	3	0	No	Yes	6	Yes
9	83	M	SCC	Leg	Inguinal	1.7 cm large 5 mm deep	1	0	No	No	15	No
10	61	F	SCC	Forearm	Axillary	Chronic scar	3	0	No	No	9	No
11	25	F	PEM	Back	Axillary Bilateral	11 mm deep	7	1	Yes	No	61	No
12	46	M	PEM	Back	Axillary Bilateral	Other skin carcinoma	3	0	No	No	41	?
13	68	M	SCC	Hand	Humeral twice axillary, cervical	3.5 cm large Poor differentiation	7	0	No	No	6	?

EBD: epidermolisis bullosa dystrophyca, LVI: lymphovascular invasion.

**Table 2:** Criteria for high-risk cutaneous squamous cell carcinoma [12, 22, 24–26].

Histopathologic factors	
Size >2 cm	
High-risk location (head and neck)	
In-transit metastatic lesion	
Poor differentiation	
Perineural invasion	
Tumour thickness >5-6 mm	
Desmoplastic growth	
Other factors	
Radiation field	
Patients with immunosuppression (transplantation and others)	
Recurrence	
Multiple SCCs	
Marjolin's ulcer (carcinoma in burn scar or chronic ulcer)	

**Table 3:** Review of studies with sentinel lymph node biopsy in patients with MCC.

Author	Reference Number	Year	patients with SLNB	H-E ± IHC	+SN	CLND	+ NSN	Nodal recurrence in SN patients	Median followup (months)
Allen et al.	[5]	2005	54	NS	12	8	2	Not detailed*	40
Maza et al.	[11]	2006	23	Both	11	8	4	2	36.1
Gupta et al.	[7]	2006	30/61	Both	7	?	—	Not detailed	—
Ortin-Perez et al.	[12]	2007	8	Both	3	3	0	0	55
Warner et al.	[13]	2008	11/17	Both	3	2	?	5	16
Shnayder et al.	[14]	2008	10/15	Both	4	1	1	1	24
Bajetta et al.	[15]	2009	21/95	NS	8	8	4		65
Fields et al.	[16]	2011	153	Both	45	21	6	8/108	41
Howle and Veness	[17]	2012	16	Both	8	3	2	2/8	19.5

Total			326		101	54	19		

*One out of 21 SN negative patients results published in a previous article (20) with a median followup of 19 months.

NS: not specified.

## Abbreviations

SLNB: Sentinel lymph node biopsy
CLND: Completion lymph node dissection
ELND: Elective lymph node dissection
MCC: Merkel cell carcinoma
SCC: Squamous cell carcinoma
PEM: Pigmented epithelioid melanocytoma
SN: Sentinel lymph node
NSN: Nonsentinel lymph node.
